# The influence of interpersonal harmony on sustainable consumption behavior in China from a Confucian perspective: exploring the dual-path mediating role of ethics and norms

**DOI:** 10.3389/fpsyg.2025.1664625

**Published:** 2025-09-26

**Authors:** Yuanlai Xin, Chun-Shuo Chen, Chaoqiao Yang

**Affiliations:** ^1^Jiangxi University of Chinese Medicine, Nanchang, China; ^2^Dhurakij Pundit University, Chinese International College, Bangkok, Thailand

**Keywords:** environmental cognition, ethical evaluation, sustainable consumption behavior, personal norms, interpersonal harmony

## Abstract

**Introduction:**

Resource scarcity is a critical issue facing the world today. Environmentally friendly consumption is essential for achieving sustainable social development. While existing literature has predominantly examined the harmony between humans and the environment, there is limited understanding of how interpersonal harmony among individuals influences sustainable consumption behavior.

**Method:**

This study collected survey data from 526 urban residents in China. Structural equation modeling was used to test the hypothesized relationships among interpersonal harmony, ethical evaluation, personal norms, and sustainable consumption behavior.

**Results:**

(1) All scales demonstrated good reliability (Cronbach’s *α* > 0.70, CR > 0.70) and validity (AVE > 0.50). CFA results confirmed acceptable model fit (Srmr = 0.038, Rms theta = 0.098). (2) Interpersonal harmony had a significantly positive effect on sustainable consumption behavior. (3) Ethical evaluation played an independent mediating role between interpersonal harmony and sustainable consumption behavior. (4) Personal norms played an independent mediating role between interpersonal harmony and sustainable consumption behavior. (5) Ethical evaluation and personal norms as chain mediators in the association between interpersonal harmony and sustainable consumption behavior. (6) Environmental cognition positively moderated the relationship between ethical evaluation and personal norms.

**Discussion:**

These findings highlight the importance of interpersonal harmony in promoting sustainable consumption behavior. Practically, the study suggests that managers and policymakers should guide consumers to cultivate harmonious cultural values and strengthen civic moral awareness to encourage sustainable consumption.

## 1 Introduction

In recent years, the concepts of environmental protection and sustainable development have gradually gained widespread acceptance (Han et al., 2025). Sustainable consumption, as an emerging consumption model, is regarded as one of the main catalysts for sustainable development (Haider et al., 2022). The significance of sustainable consumption in its economic, social, and environmental impacts has attracted considerable attention from academic circles (Aktan and Kethüda, 2024). The antecedents of sustainable consumption behavior are diverse (Mondal and Samaddar, 2022), with scholars discussing factors such as lifestyle (Li et al., 2022) and external motivations (Sharma et al., 2022). However, the environmental decision-making process is inevitably influenced by the social environment and socio-cultural values (Zhao et al., 2024).

Since Hofstede introduced the cultural dimension theory into the mainstream management theory system in 1980, differences in national cultural values and their impacts have received extensive attention in the management field. However, the concept of culture is too broad, and existing literature generally focuses on cultural values for research. At present, research on the impact of cultural values on sustainable behaviors has limitations (Chwialkowska et al., 2024), particularly in the following three aspects: (1) Over the past two decades, many studies examining the impact of cross-cultural values on consumer behavior have largely been based on the value research by Hofstede (1980), Schwartz (1994), and Stern (2000). Scholars have recently questioned whether measurement tools derived from Western values are applicable to research in Eastern cultures (Ye et al., 2018). (2) In addition to values closely linked to nature, other cultural values also deserve further investigation (Halkos and Matsiori, 2017; Kim and Stepchenkova, 2020). Human development is also closely related to social harmony and progress (Xu et al., 2022). Although more attention has been given to the harmony between humans and the environment, the harmony between people has been somewhat neglected. (3) Consumers may claim to care about ethics but fail to act accordingly (Huang and Yang, 2023). Individuals increasingly recognize the moral responsibility to protect and preserve the environment for future generations.

The main objectives of this study, based on the aforementioned practical and theoretical backgrounds and actual data analysis, are as follows: (1) This study aims to expand the antecedent variables of sustainable consumption behavior and refine theoretical research on interpersonal harmony values from the Confucian cultural perspective. Meanwhile, this study seeks to address the limitations stemming from differences in measurement tools for Chinese and Western cultural values, thereby filling the gap in quantitative research on Chinese indigenous cultural values (Zhao et al., 2024). China is deeply influenced by Confucian culture, which exhibits strong social attributes in the Chinese social context and helps explain interpersonal and social harmony orientations (Li and Zhong, 2006; Lun and Miu, 2012). (2) This study seeks to enrich the research conclusions of value-belief-norm theory and construct a new model based on this theory, to explore the dual mediating roles of ethical evaluation and personal norms in the relationship between interpersonal harmony and sustainable consumption behavior. (3) This study broadens the theoretical field of interpersonal harmony in relation to sustainable consumption behavior. By introducing environmental protection knowledge as a moderating variable, this study explores its moderating effect between ethical evaluation and personal norms from the perspective of environmental protection knowledge. This study attempts to reveal the complex relationship between interpersonal harmony and sustainable consumption behavior from a Confucian perspective, while aiming to provide theoretical support and empirical evidence to promote sustainable consumption and hopes to offer new perspectives for businesses and policymakers.

## 2 Literature review and hypothesis development

### 2.1 Theoretical framework

#### 2.1.1 Manifestations of culture at different levels of depth

**Figure 1:**
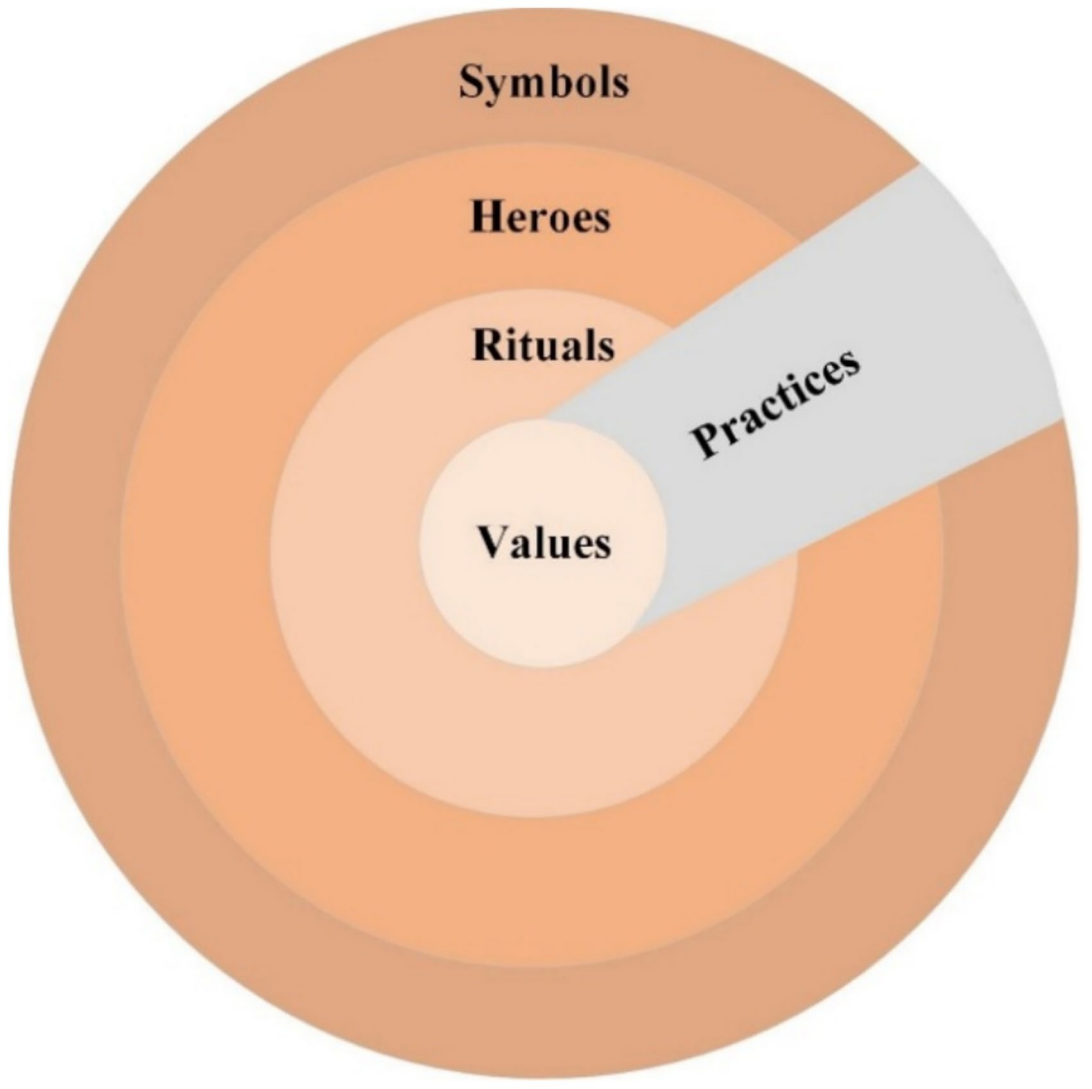
The “Onion Diagram”: manifestations of culture at different levels of depth.

As shown in Figure 1, culture encompasses symbols (words, gestures, and images with specific meanings), heroes (living or deceased, real or fictional figures), and rituals (collective activities). In the “Onion Diagram,” the outer layer of culture can change rapidly, but the core part representing culture (i.e., values) changes very slowly (Hofstede, 1980). Confucian culture has been identified as one of the 10 cultural clusters in global research (Gupta et al., 2002). Confucian cultural is the most important part of China’s overall worldview (Hofstede, 1980; Lau et al., 2021); it is not merely a relic of the past but also shapes Chinese values and exerts a profound influence on Chinese people’s lifestyles and consumption patterns (Xu et al., 2022; Terpstra-Tong and Ralston, 2025). As illustrated in the aforementioned onion model (Hofstede, 1980), this study posits that Confucian culture exhibits a multidimensional structure. Interpersonal harmony as Confucian culture core value (Chen et al., 2015), and serves as one of the moral standards in Confucian culture (Han and Altman, 2010). This study thus focuses exclusively on interpersonal harmony values on Chinese people’s behavior from a Confucian cultural perspective.

#### 2.1.2 Value–belief–norm theory

**Figure 2:**
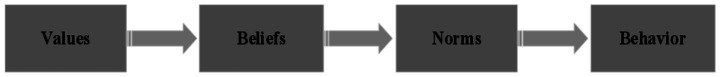
Value–belief–norm (VBN) theory.

Gardner and Stern (1996) proposed value–belief–norm (VBN) theory, as illustrated in Figure 2. This theory primarily consists of three components: values, specific beliefs, and individual norms. Specifically, values determine ecological worldviews (i.e., the new environmental paradigm), which represent an individual’s general view of the natural environment (Dunlap et al., 2000) and reflect a new perspective on the relationship between humans and nature. The ecological worldview directly influences awareness of consequences, in turn triggering the sense of responsibility, ultimately activating personal norms (Stern et al., 1999). When individuals are convinced that their actions will result in corresponding consequences and are willing to take responsibility for those consequences, their behaviors tend to align with their personal norms (Gkargkavouzi et al., 2019). VBN theory emphasizes that values and personal norms are key variables in explaining environmental behavior (Zeiske et al., 2021). The causal pathway follows the sequence of “value → belief → norm → environmental behavior” (Van der Werff and Steg, 2016). This theory has been widely applied in research on pro-environmental behavior (Zeiske et al., 2021).

However, this theory has three key areas that warrant further exploration: (1) Most existing studies based on this theory focus on biosphere values as the primary predictor of sustainable behavior (Navarro et al., 2017). However, relying solely on the predictive power of biosphere values may not be sufficient to explain the personal motivations for sustainable practices. The subset of values influencing sustainable purchase behavior also varies across cultures (Sharma and Jha, 2017). (2) Although most empirical studies primarily analyze path relationships between upper and lower-level variables (Fornara et al., 2016; Whitley et al., 2016), few have drawn conclusions regarding partial or full chain mediation. (3) The concept and scales of altruism stem from Western research, while interpersonal harmony in Eastern Confucian culture also advocates caring for the well-being and development of others. In the field of sustainable development, scholars have shown through preliminary studies that consumers’ pro-environmental behaviors do vary across countries and cultures (Rahman et al., 2023). Chinese people are deeply influenced by Confucian culture (Terpstra-Tong and Ralston, 2025). Chinese individuals tend to avoid extreme behaviors (Lu et al., 2017) and are more concerned with interpersonal harmony within social relationships (Farh et al., 2004). The present study therefore builds on VBN theory and develops a chain mediation model that examines the impact of interpersonal harmony values on sustainable consumption behavior from a Confucian cultural perspective. The model developed in this study can effectively serve as a complementary investigation into the three aforementioned theoretical extensions of Value–belief–norm theory.

### 2.2 Hypotheses development

#### 2.2.1 Relationship between interpersonal harmony and sustainable consumption behavior

Cultural or personal values exert a powerful influence on consumer behavior (Che and Zhou, 2025; Sharma and Jha, 2017), and consumers from different cultural backgrounds engage in consumption behaviors that vary accordingly (Duong et al., 2024). Many behavioral norms in China stem from Confucian culture. Individuals with higher levels of interpersonal harmony are more likely to prioritize the goals of the collective over their own (Kim and Choi, 2005; Leonidou et al., 2010). They consciously or unconsciously place group interests above individual interests, and protecting the environment benefits the group’s prosperity (Cho et al., 2013). Pro-environmental behaviors also serve to project a positive image and social identity (Wu et al., 2019). Individuals with a higher level of interpersonal harmony are more likely to engage in sustainable consumption behaviors. Furthermore, individuals with higher interpersonal harmony tend to imagine other’s feelings. In other words, they more easily exhibit a sense of empathy, specifically empathy toward others and concern for unfortunate others. They also tend to spontaneously adopt others’ psychological perspectives (Di Fabio and Tsuda, 2018). Highly empathetic individuals are more likely to experience a stronger connection to nature. People with a stronger connection to nature tend to perceive themselves as part of a broader natural community. They view their own well-being as intertwined with the well-being of the natural world (Di Fabio and Kenny, 2021). For instance, Schmitt et al. (2018) investigated the involvement of consumers with 39 pro-environmental behaviors and found that individuals with high interpersonal harmony tend to exhibit life satisfaction, and this life satisfaction correlates positively with most pro-environmental behaviors.

Therefore, individuals with higher interpersonal harmony are more likely to consider their responsibilities to others, society, and future generations, making them more prone to engage in sustainable consumption behaviors.

*H1*: Interpersonal harmony positively influences sustainable consumption behavior.

#### 2.2.2 The relationship between interpersonal harmony and ethical evaluation

Consumers tend to engage in ethical evaluations before deciding to execute certain behaviors (Chan et al., 2008). A consumer’s ethical evaluation influences their cognition and subsequent behaviors (Wang et al., 2023). Define moral obligation evaluations as individuals’ judgments of good and evil, right and wrong, based on their values. Given that every person holds different values, their moral standards vary accordingly. Wu et al. (2019) suggest that interpersonal harmony can positively influence employees’ pro-environmental behavior through a sense of accountability. In the face of conflicts, individuals with higher interpersonal harmony tend to adopt integrative or cooperative strategies. A reasonable inference is that consumers with higher interpersonal harmony, guided by the people-oriented thought, will pay more attention to themselves and others. In this culture, immoral behaviors are more severe than individualistic consumption behaviors and are even more unacceptable (Bernardi and Long, 2004; Mo et al., 2023). Sustainable consumption, which serves the environment, sustainable development, and social responsibility, is beneficial to the development of all humanity, working for the self, others, and the society (Jaeger, 2021). Hence, individuals with higher interpersonal harmony contribute to building honest and mutually beneficial social interactions, focusing more on the interests of themselves and their community. They are more engaged with public and environmental causes, thus leading to higher ethical evaluations of sustainable consumption behaviors.

*H2*: Interpersonal harmony positively influences ethical evaluation.

#### 2.2.3 The relationship between ethical evaluation and sustainable consumption behavior

Vitell and Muncy (1992) defined consumer ethical evaluation as “the moral principles and standards that guide individuals or groups in the acquisition, use, and disposal of goods and services.” Before performing a specific action, individuals conduct a ethical evaluation based on their values. This evaluation typically does not rely solely on pre-action obligation evaluation but integrates obligations and objectives, ultimately forming a moral judgment of the action (Hunt and Vitell, 1986). Pre-action obligation evaluations influence individuals’ behavioral preferences. When a specific action is perceived as morally correct and in line with ethical principles, individuals with high levels of obligation evaluation tend to view the action as more in accordance with their principles, and they feel a moral obligation to carry out the behavior, leading action (Sweeney et al., 2010). When an action is perceived as beneficial to society and yields positive and pleasant consequences, individuals with high levels of teleological evaluation are more likely to execute the behavior (Yin et al., 2016). The connection between moral ideology and sustainable consumption seems evident because consumers are increasingly aware that sustainable consumption is an aspect of environmental issues (Rahman et al., 2020). Therefore, driven by personal moral obligations toward the environment, consumers may engage in eco-friendly purchasing behavior (Balderjahn et al., 2013). Sustainable behavior is a form of social behavior. It not only involves the purchase of socially responsible brands but also engages in charitable donations and environmentally beneficial actions (Rahman et al., 2023). Thus, when individuals have positive pre-action ethical evaluations of sustainable consumption, they believe that sustainable consumption is morally correct, and the consequences and objectives of the behavior are positive. They are therefore more likely to embrace and implement sustainable consumption behaviors.

*H3*: Ethical evaluation positively influences sustainable consumption behavior.

#### 2.2.4 The mediating role of ethical evaluation

Morality is deeply influenced by cultural backgrounds, and moral choices can vary slightly across different cultures (Hasan et al., 2023). Consumers’ ethical evaluations affect their cognition and subsequent behaviors (Wang et al., 2023). Feng and Wang (2023) found, from a perspective of traditional Chinese culture, that Chinese consumers care more about the “public self,” which is closely connected to the social environment. Li and Shao (2023) emphasized the individuals’ moral obligations to perform a certain behavior serve as the driving force for altruistic actions. This finding demonstrates that ethical evaluation plays a connecting role between cultural values and subsequent behaviors. Existing research also supports this view because Qiu (2016) showed that in the factors influencing tourists’ willingness to engage in civilized tourism behaviors, moral norms partially mediate the relationship. Steg et al. (2005) suggested that whether an individual feels morally obligated to act in an environmentally friendly manner determines their choice of environmentally-friendly transportation.

*H4*: Ethical evaluation mediates the relationship between interpersonal harmony and sustainable consumption behavior.

#### 2.2.5 The relationship between interpersonal harmony and personal norms

The relationship between values and personal norms has been confirmed by most scholars (Nordlund and Garvill, 2003). Hou et al. (2014) studied the new generation of employees with good interpersonal harmony and found that this harmonious work value helps them maintain a positive self-view. When employees work in ways that align with their positive self-view, a sense of self-confidence will be created, which can lead to interpersonal altruism in the workplace and further promote pro-social behaviors of employees. Chen et al. (2022) indicated that interpersonal harmony represents an optimistic, open attitude that helps stabilize interpersonal relationships and plays a critical role in resolving workplace interpersonal conflicts (Chen and Li, 2009; Zhang et al., 2004). Therefore, individuals with higher interpersonal harmony tend to engage in behaviors that align with their positive self-views, leading to altruism, in turn positively influencing personal norms (Guan and Zhang, 2023) Interpersonal harmony enhances interpersonal relationships and communication, and good communication has a significant impact on personal norms (Han and Xu, 2020); Dunlop et al. (2008) also found that interpersonal communication regarding health issues influences people’s perceptions of health risks and corresponding behaviors. If these effects are strong enough, interpersonal communication can influence final behavior by altering individuals’ normative judgments (Kahlor, 2007). Individuals who place greater emphasis on interpersonal information are more likely to enhance their personal norms (Wang et al., 2023).

*H5*: Interpersonal harmony positively influences personal norms.

#### 2.2.6 The relationship between personal norms and sustainable consumption behavior

When individuals have strong personal norms based on experience, they may exhibit pro-environmental behavior in a moral sense (Shang and Wu, 2022). At present, two paths lead from personal norms to pro-environmental behavior: One path is that following personal norms leads to feelings of pride, enhanced self-esteem, sense of security, or other favorable self-evaluations. Most researches on environmental issues related to car emissions suggest that individuals who are aware that their use of cars contributes to environmental problems are more likely to feel responsible for reducing car usage to help solve these issues than those who lack this awareness (Moser, 2015). Parker (2009) pointed out that to achieve sustainable consumption behavior, people manage their self-presentation, and adhering to personal norms is a common way to enhance self-identity (Aagerup and Nilsson, 2016). The other path is that violating personal norms leads to feelings of guilt, self-deprecation, loss of self-esteem, or other negative self-evaluations. Individuals tend to transform these negative feelings. When a person feels responsible for violating moral standards or principles, realizing that they have not acted correctly—for example, purchasing products that severely harm the environment—can cause consumers to feel guilty, triggering emotional distress and possibly evoking negative anticipatory emotions (Steenhaut and Van Kenhove, 2006). On the other hand, adopting eco-friendly products can be seen as a way to relieve personal discomfort and generate positive emotions. Purchasing from manufacturers whose products and processes are more environmentally friendly can enhance an ideal self-concept and make consumers “feel good” (Pickett-Baker and Ozaki, 2008). Therefore, environmentally responsible behavior therefore depends on the strength of personal norms concerning such behaviors (Thøgersen, 2006). A large number of studies shows a positive correlation between personal norms and pro-environmental behavior, such as actions to reduce PM2.5 (Ru et al., 2019), green travel (Han et al., 2019), green lodging (Han and Hyun, 2018), visiting environmental responsibility museums (Han and Hyun, 2017), and the willingness to pay for park conservation.

*H6*: Personal norms positively influence sustainable consumption behavior.

#### 2.2.7 The mediating role of personal norms

VBN Theory emphasizes that values and personal norms are key variables in explaining environmental behavior (Zeiske et al., 2021). Values influence personal norms, in turn affecting behaviors like reducing personal car usage (Nordlund and Garvill, 2003). People who prioritize interpersonal harmony tend to put the group’s goals above their own (Kim and Choi, 2005; Leonidou et al., 2010), and they are generally more concerned with public interests, including the environment. When individuals are inclined to engage in behaviors consistent with a positive self-view, altruism is often generated, potentially positively influencing personal norms (Guan and Zhang, 2023). Following personal norms typically results in feelings of pride or other favorable self-evaluations because adherence to personal norms is a common way to enhance self-identity (Aagerup and Nilsson, 2016). Environmental norms are rules commonly accepted within a group that restrict harmful environmental behaviors or specify environmentally friendly actions (Mohr, 1994). People who place more importance on interpersonal harmony will further promote their environmental norms to encourage the implementation of pro-environmental actions (Wang et al., 2023).

*H7*: Personal norms mediate the relationship between interpersonal harmony and sustainable consumption behavior.

#### 2.2.8 The relationship between ethical evaluation and personal norms

This study posits that for ethical evaluation to activate personal norms, two conditions must be met. First, before performing a specific action (in this study, pro-environmental behaviors related to sustainable consumption), consumers will conduct a pre-action ethical evaluation based on their own values (Barbarossa et al., 2015). Individuals must consider the consequences of their actions before experiencing responsibility (DeGroot and Steg, 2009). Second, the higher an individual’s ethical evaluation, the higher their sense of responsibility, thereby activating their personal norms (Ateş, 2020). Ethical evaluation influences an individual’s moral preferences; when a particular behavior is considered correct and in line with moral principles, they feel a moral responsibility to engage in that behavior (Sweeney et al., 2010). Personal norms are internal standards that guide individuals, specifically related to sustainable behaviors (Li and Zhang, 2023), whereby individuals feel responsible for meeting their own environmental expectations (Chou, 2014). Given that pro-environmental behaviors are morally right and socially desirable, individuals with high levels of moral (deontological) evaluation are more sensitive to moral and environmental issues (Wang et al., 2023). They are more likely to believe that the natural environment has moral rights to be properly treated and that people have a moral responsibility to protect the environment (Leonidou et al., 2010). Therefore, when an individual believes that sustainable consumption is consistent with their moral standards and recognizes its potential positive consequences, they feel a moral responsibility to engage in such behavior.

*H8*: Ethical evaluation positively influences personal norms.

#### 2.2.9 The chain mediating role of ethical evaluation and personal norms

VBN theory can effectively explain this relationship (Zeiske et al., 2021). According to this theory, values follow a pathway of “values → beliefs → norms → environmental behavior” (Van der Werff and Steg, 2016). As Steg et al. (2005) argued, when environmental consequences awareness and environmental responsibility attribution can predict specific behaviors, the predictive ability of the VBN model is strengthened. Therefore, when individuals have strong awareness of consequences, their ethical evaluation of sustainable consumption will be higher. This ethical evaluation activates personal norms, with personal norms being the main direct predictor of pro-environmental behavior (Gkargkavouzi et al., 2019).

*H9*: Ethical evaluation and personal norms mediate the relationship between interpersonal harmony and sustainable consumption behavior in a chain manner.

#### 2.2.10 The moderating effect of environmental cognition

Environmental cognition refers to the information individuals possess about the relationship between humans and the environment, which is accumulated through daily life experiences (Kwon et al., 2016). Environmental cognition prompts individuals to identify and confirm information related to environmental issues, in turn enabling consumers to transform this knowledge into a sense of moral responsibility. The indirect influence of environmental cognition on people’s environmental awareness may be greater than its direct influence (Kollmuss and Agyeman, 2002), helping consumers form a clearer understanding of the causes of ecological crises and thus providing explanatory power for their willingness to engage in sustainable consumption (Kwon et al., 2016). A reasonable inference is that as consumers’ understanding of environmental conditions becomes clearer, the environmental cognition they have accumulated will lead to a more precise understanding of the consequences and solutions for environmental protection. This knowledge accelerates the transition from ethical evaluation to personal norms, driving consumers to activate their personal norms. Consumers with more knowledge about green purchasing perceive higher value in green consumption and are more capable of social observation (Buenstorf and Cordes, 2008), leading them to favor eco-friendly behaviors (Wang et al., 2021). Lin and Niu (2018) argued that sustainable consumption is driven by consumers’ deeper environmental cognition, stronger moral sense, and greater sense of responsibility, with environmental cognition positively affecting their environmental attitudes and behaviors. This knowledge and sense of responsibility eventually lead to a sense of well-being, allowing individuals to define positive personal norms (Law et al., 2017). Therefore, when individuals have higher ethical evaluations of sustainable consumption, those with greater environmental cognition are more likely to translate ethical evaluations into personal norms. This study proposes the following hypothesis:

*H10*: Environmental cognition positively moderates the relationship between ethical evaluation and personal norms.

**Figure 3:**
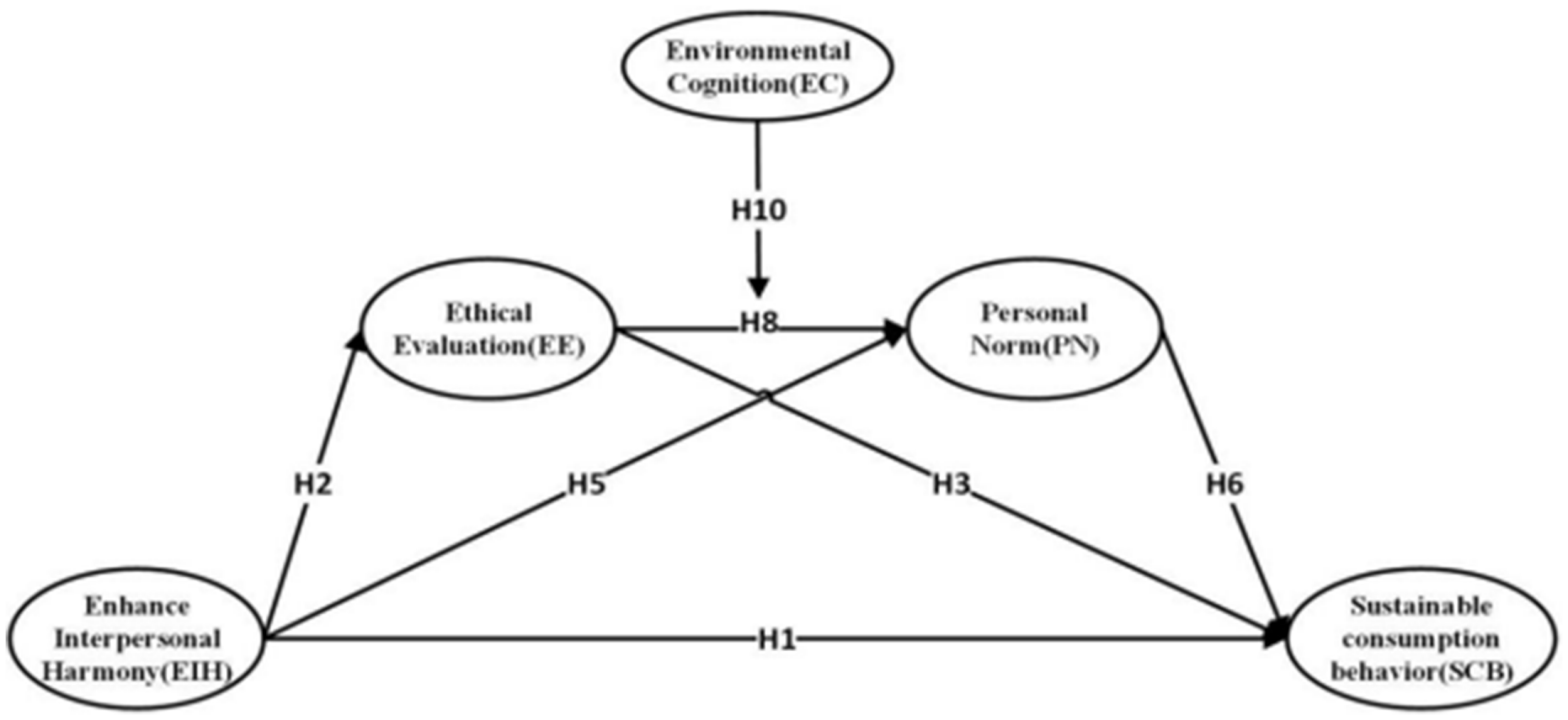
Theoretical Framework. H4: Enhance Interpersonal Harmony (EIH) → Ethical Evaluation (EE) → Personal Norm (PN). H7: Enhance Interpersonal Harmony (EIH) → Personal Norm (PN) → Sustainable consumption behavior (SCB). H9: Enhance Interpersonal Harmony (EIH) → Ethical Evaluation (EE) → Personal Norm (PN) Sustainable consumption behavior (SCB).

The research model of this study is presented in Figure 3.

## 3 Method

### 3.1 Measurement of variables

#### 3.1.1 Latent variables

The scale for interpersonal harmony is based on Chen et al. (2015) with 12 items. Sustainable consumption behavior is measured using the scale from Quoquab et al. (2019), with 24 items. The ethical evaluation scale follows Wang et al. (2023) with 7 items. Personal norms are measured using Li and Zhang (2023) scale, consisting of 3 items. Environmental cognition is assessed using the scale from Wang et al. (2016), with 5 items designed to indirectly measure individuals’ environmental cognition.

#### 3.1.2 Control variables

The socioeconomic characteristics of the sample included gender, age, educational level, and monthly income as control variables.

#### 3.1.3 Measure items

With regard to these variables, namely interpersonal harmony, ethical evaluation, personal norms, environmental cognition, and sustainable consumption behavior, their measurement items are selected from the mature scales in previous literature. As most of the original scales were developed in English and applicable to English-speaking countries, a back translation process is adopted to edit the survey. The survey consists of three main sections: the first section provides open-ended explanations; the second section is the main body of the questionnaire, which comprises a scale consisting of survey questions; and the third section includes demographic variables. All scales are measured using a five-point Likert scale, where 1 means strongly disagree, 2 means disagree, 3 means neutral, 4 means agree, and 5 means strongly agree.

### 3.2 Sampling technique and data collection

In alignment with the objectives of this study, the target population comprises urban residents in China, specifically individuals who have resided in or lived within urban or town areas in China for over six months in the past year. The selection is made for the following reasons: first, compared with rural consumers, urban consumers dominate sustainable consumption (Wang et al., 2016), possessing higher levels of environmental and sustainability knowledge (Moser, 2016), ensuring a better understanding of sustainable consumption when filling out the questionnaire, thereby guaranteeing the authenticity of responses. Second, urban residents typically possess a higher level of personal cultivation, aiding in more effective cooperation with the survey process, thereby enhancing the quality of the data collected for this study.

From January 12 to March 28, 2024, questionnaires were distributed in nine China-based CR Vanguard, Wanjia Mart, and Olé supermarkets, primarily through links in membership benefit WeChat groups or product promotion WeChat groups. Although distributing questionnaires through WeChat groups allows for rapid access to potential samples, the quality of responses in an online environment is difficult to effectively control (Reips, 2021). To ensure data quality, a reverse lie detection item 37, “I will not buy environmentally friendly products,” was included as item 14 in the questionnaire, alongside the direct item “I purchase and use products which are environmentally friendly.” If responses to these items were inconsistent, they were flagged as abnormal and excluded from the analysis. While such a design can effectively identify inattentive respondents (e.g., those who randomly select options or fail to read the question content), it may also result in a high elimination rate due to strict exclusion criteria. Ultimately, a total of 1,277 responses were collected, and after excluding invalid responses (e.g., duplicate answers, illogical patterns), 526 valid responses remained, resulting in a valid response rate of 41.19%.

### 3.3 Data analysis method

To assess the significance of the model, PLS–SEM is employed to analyze the hypothesized relationships in this study. This method is widely used for testing new research trends and developing models, rather than merely confirming existing theories (Urbach and Ahlemann, 2010). Furthermore, PLS–SEM is chosen for its ability to address measurement errors in the structural model while estimating causal relationships between latent constructs A two-step analysis is conducted to test these hypotheses.

## 4 Empirical analysis

### 4.1 Demographic characteristics and descriptive statistics

#### 4.1.1 Demographic characteristics statistics

As shown in Table 1, the highest number of respondents by gender is women, with 320 respondents, accounting for 60.8%, which is higher than the number of male respondents. This can be attributed to the fact that the survey was distributed to supermarket members, and women are more likely to register for supermarket membership than men. Regarding age, the largest proportion of respondents is from the 18–29 age group, accounting for 39.5%. Individuals under 18 were excluded due to the lack of disposable income. In terms of education, the highest frequency is at the university undergraduate level (33.4%), while the lowest proportion is at the doctoral level (2.4%). As for marital status, the highest frequency is married, with 297 respondents, accounting for 56.4%. Regarding occupation, “other” is the highest category with 167 respondents, accounting for 31.7%. The range of income with most number of respondents is 5,001–7,500 RMB, comprising 31.94%. These findings suggest that the data collected from the survey are reasonable and suitable for further analysis (Table 2).

**Table 1:** Survey respondent characteristics.

Name of demographiccharacteristics	*n*	%	Name of demographiccharacteristics	*n*	%
Gender			Marital status		
Male	206	39.163	Unmarried	229	43.536
Female	320	60.837	Married	297	56.464
Age group			Education level		
18 ~ 29	208	39.544	High school and below	93	17.681
30 ~ 39	172	32.700	College degree	139	26.426
40 ~ 49	97	18.441	Bachelor’s degree	176	33.460
50 ~ 59	37	7.034	Master’s degree	105	19.962
60 and above	12	2.281	Doctorate	13	2.471
Monthly income			Occupation type		
≤ 2,500 RMB	58	11.027	Worker/Service	81	15.399
2,501–5,000 RMB	115	21.863	Engineer/Technician	46	8.745
5,001–7,500 RMB	168	31.939	Personnel in the fields of science, education, culture, and health	120	22.814
7,501–10,000 RMB	95	18.061	Other	167	31.749
10,001–12,500 RMB	48	9.125			
≥12,501 RMB	42	7.985			

**Table 2:** Descriptive statistics.

Constructs	Mean	Standard deviation	Skewness	Kurtosis
SCB	3.445	0.645	0.036	−0.391
EH	3.614	0.946	−1.026	0.540
PN	3.569	1.084	−1.004	−0.089
EE	3.711	0.969	−0.773	0.174
EC	3.718	1.058	−1.001	0.154

#### 4.1.2 Descriptive statistics

The mean value of Sustainable Consumption Behavior (SCB) was 3.445, slightly above the scale midpoint, indicating that respondents generally exhibited a moderately positive tendency toward sustainable consumption. The mean score of Enhancing Interpersonal Harmony (EIH) stood at 3.614, reflecting that respondents attached considerable importance to interpersonal harmony in the given context. Personal Norm (PN) yielded a mean of 3.569, suggesting that respondents’ moral responsibility toward sustainable consumption was at a moderate level. Ethics Evaluation (EE) recorded the highest mean value among all variables at 3.711, demonstrating that respondents generally recognized the ethical correctness of sustainable consumption. Environmental Cognition (EC) presented a mean of 3.718, which was close to that of EE, indicating that respondents had a relatively high self-perceived level of attention to and understanding of environmental issues. Regarding standard deviation (SD) results: All variables exhibited SD values ranging from [0.645 to 1.084], indicating a certain degree of heterogeneity in scale responses. Particularly, Personal Norm (PN) showed the highest degree of dispersion (SD = 1.084), suggesting significant differences in moral responsibility perception among different respondents. Analysis of skewness and kurtosis results revealed that except for SCB (skewness = 0.036), all other variables demonstrated negative skewness values [−0.773 to −1.026], indicating slight left skewness of the data (responses clustered at the “agree” end). Kurtosis values all fell within the range of ±0.5, meeting the robustness requirements for data distribution in PLS–SEM analysis.

### 4.2 The measurement model

In this study, Smart PLS were used in the statistical analysis of the questionnaire survey data. Cronbach’s alpha coefficients were used to test the internal consistency of the scales and ensure their reliability (Cronbach and Meehl, 1955). Composite reliability (CR), average variance extracted (AVE), the square root of AVE, and Pearson correlation coefficients among latent variables were used to conduct the validity tests. HTMT ratios examine potential multi-collinearity (Kautish et al., 2022). PLS–SEM is chosen for its ability to address measurement errors in the structural model while estimating causal relationships between latent constructs a two-step analysis is conducted to test these hypotheses (Table 3).

**Table 3:** Items, Cronbach’s alpha, standard loading, composite reliability (CR), and average variance extracted (AVE).

Constructs	Cronbach’s alpha	CR	AVE
EH	0.935	0.944	0.583
SCB	0.960	0.963	0.521
EE	0.914	0.931	0.659
PN	0.867	0.919	0.790
EC	0.906	0.929	0.725

The validity of the scales is assessed using convergent and discriminant validity in this paper. Convergent validity is acceptable if the average variance extracted (AVE) is greater than 0.500 and composite reliability (CR) exceeds 0.700 (Campbell and Fiske, 1959). Table 4 indicates that the square roots of the AVE of seven variables are higher than the correlation coefficients between any two variables, indicating qualified discriminant validity (Koufteros, 1999). Structural equation modeling (SEM) is also used to evaluate the fit of the model and its indices (Cronbach and Meehl, 1955).

**Table 4:** Discriminant validity—Fornell–Larcker criterion.

	EC	EE	EH	PN	SCB
EC	0.851				
EE	0.137	0.812			
EH	0.112	0.435	0.764		
PN	0.127	0.377	0.443	0.889	
SCB	0.112	0.455	0.552	0.460	0.722

To check for potential multi-collinearity, the HTMT ratios for all constructs are examined, as shown in Table 5. The HTMT ratios are found to be below 0.850, meeting the required threshold (Kautish et al., 2022).

**Table 5:** HTMT (heterotrait–monotrait ratio).

	EC	EE	EH	PN	SCB
EC					
EE	0.152				
EH	0.120	0.469			
PN	0.141	0.421	0.488		
SCB	0.118	0.483	0.575	0.500	

### 4.3 The measurement model

To assess the significance of the model, PLS–SEM is employed to analyze the hypothesized relationships in this study. This method is widely used for testing new research trends and developing models, rather than merely confirming existing theories (Urbach and Ahlemann, 2010).

First, the path coefficients for all structural relationships are estimated to examine direct and moderating effects. As shown in Table 6, the path analysis reveals the following significant relationships: Interpersonal harmony has a significant positive effect on sustainable consumption behavior (*b* = 0.361, *p* = 0.000 < 0.001). This finding indicates that individuals with a higher tendency toward interpersonal harmony are more likely to engage in sustainable consumption behaviors, thus supporting H1. Interpersonal harmony has a significant positive effect on ethical evaluation (*b* = 0.435, *p* = 0.000 < 0.001), supporting H2. Ethical evaluation has a significant positive effect on sustainable consumption behavior (*b* = 0.216, *p* = 0.000 < 0.001), confirming H3. Interpersonal harmony has a significant positive effect on personal norms (*b* = 0.331, *p* = 0.000 < 0.001), supporting H5. Personal norms have a significant positive effect on sustainable consumption behavior (*b* = 0.218, *p* = 0.000 < 0.001), supporting H6. Ethical evaluation has a significant positive effect on personal norms (*b* = 0.218, *p* = 0.000 < 0.001), supporting H8. The interaction between ethical evaluation and environmental cognition is significant (*b* = 0.085, *p* = 0.003 < 0.005). This suggests that environmental cognition enhances the positive effect of ethical evaluation on personal norms, thus supporting H10.

**Table 6:** Direct effect testing.

Path	Path coefficient	SD	*T*	*P*	Hypotheses findings	
EH - > SCB	0.361***	0.046	7.914	0.000	H1	Support
EH - > EE	0.435***	0.049	8.843	0.000	H2	Support
EE - > SCB	0.216***	0.043	5.069	0.000	H3	Support
EH - > PN	0.331***	0.053	6.222	0.000	H5	Support
PN - > SCB	0.218***	0.049	4.477	0.000	H6	Support
EE - > PN	0.218***	0.051	4.271	0.000	H8	Support
EE*EC - > PN	0.085**	0.028	3.007	0.003	H10	Support

### 4.4 Chain mediating effect test

The indirect effect (Table 7) indicates that the path of interpersonal harmony → ethical evaluation → sustainable consumption behavior passes the significance test when *p* value is at the 0.001 level, indicating that ethical evaluation mediates the relationship between interpersonal harmony and sustainable consumption behavior. Therefore, H4 is further supported. Similarly, the path of interpersonal harmony → personal norms → sustainable consumption behavior passes the significance test at the 0.01 level, showing that personal norms mediate the relationship between interpersonal harmony and sustainable consumption behavior. Therefore, H7 is further supported. Finally, the path from interpersonal harmony to ethical evaluation to personal norms to sustainable consumption behavior is significant at the 0.01 level (*p* < 0.01). This finding indicates that ethical evaluation and personal norms play a mediating role in the effect of interpersonal harmony on sustainable consumption behavior. Specifically, ethical evaluation and personal norms act as a chain mediator between interpersonal harmony and sustainable consumption behavior, thereby supporting H9.

**Table 7:** Mediating effect testing.

Path	Path coefficient	SD	*T*	*P*	Hypotheses findings	
EH - > EE - > SCB	0.094***	0.022	4.363	0.000	H4	Support
EH - > PN - > SCB	0.072**	0.021	3.470	0.001	H7	Support
EH - > EE - > PN - > SCB	0.021**	0.007	2.945	0.003	H9	Support

### 4.5 Testing results of research hypotheses

**Figure 4:**
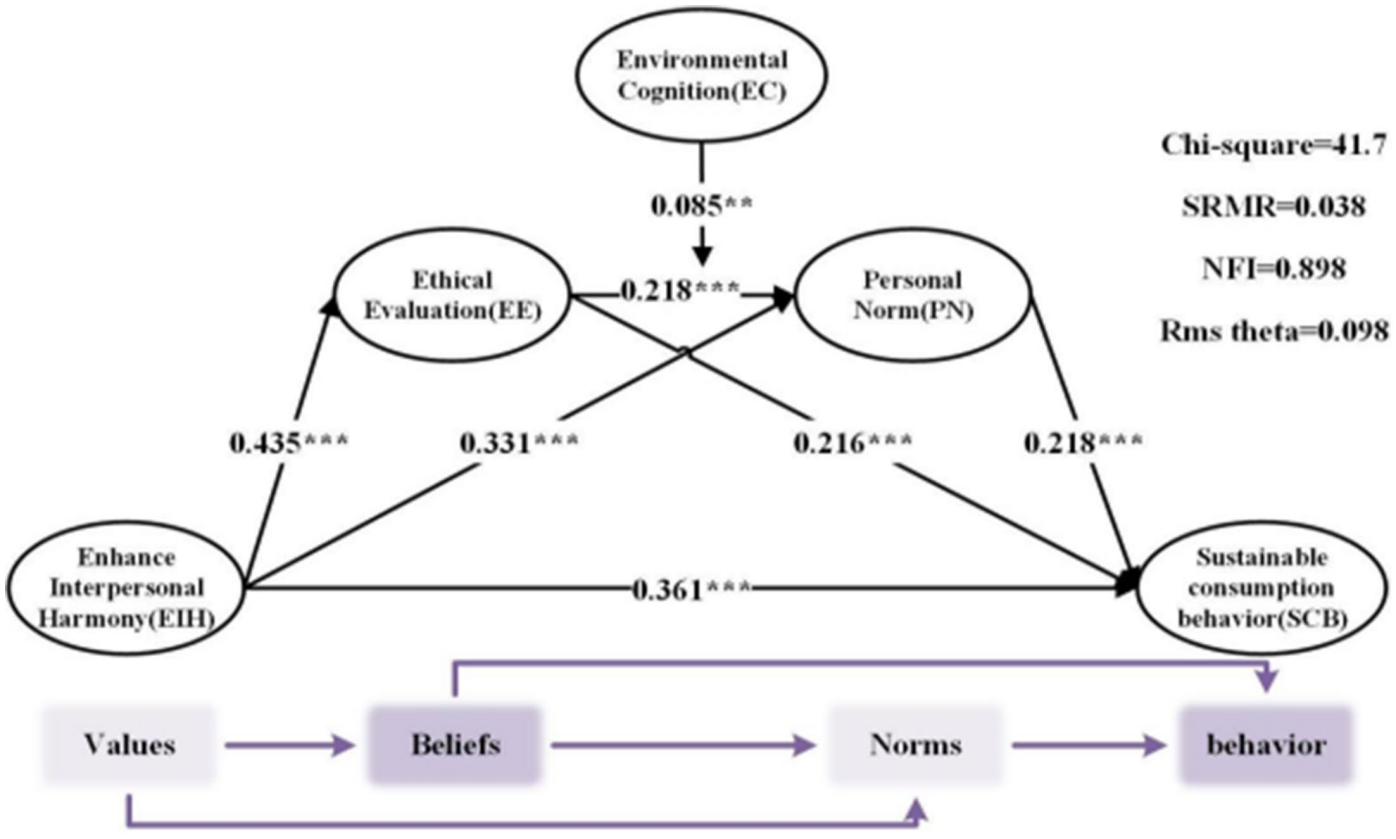
SEM of theoretical framework.

This study proposed a total of 10 research hypotheses. Statistical analysis was conducted using PLS analysis software on questionnaire reliability and validity, internal consistency of the measurement model assessment (reliability and validity tests), and other aspects, with results generally meeting the requirements. Further analysis of the direct, mediating, and moderating effects in hypothesis testing revealed that all hypotheses were supported, as shown in Figure 4 below.

## 5 Discussion

Cultural values are a key factor in predicting consumer behavior (Xin et al., 2024). Individuals from different countries may have unique perspectives and attitudes toward the same issue (Duong et al., 2024). This study interprets consumer psychological changes from the perspective of interpersonal harmony values, addressing how moral and normative constructs can enhance sustainable consumption behavior, while also revealing the boundary role of environmental cognition. The following conclusions are drawn:

(1) Interpersonal harmony values have a positive influence on sustainable consumption behavior. The findings of this study align with the views of Wu et al. (2019), suggesting that interpersonal harmony values have a significant indirect impact on green behaviors in the workplace. Zhao et al. (2024) argued that cultural values shape identity, enabling individuals to present a positive image of themselves to others through sustainable consumption choices. Interpersonal harmony facilitates the formation of collectivism (Leung et al., 2002), and extensive research confirms the positive correlation between collectivism and prosocial behaviors.
(2) Ethical evaluation plays a partial mediating role between interpersonal harmony and sustainable consumption behavior. Sustainable consumption is a behavior unique to environmental protection, sustainable development, and social responsibility, benefiting the development of humanity and serving the interests of oneself, others, and society (Jaeger, 2021). Consumers can only exhibit sustainable intentions if they perform proactive ethical evaluations of sustainability actions (Yin et al., 2016), thus activating their moral principles (Tomșa et al., 2021).
(3) Personal norms serve as a partial mediator between interpersonal harmony and sustainable consumption behavior. This conclusion is consistent with Ru et al. (2019) and Han et al. (2019), who suggest that environmentally responsible behaviors depend on the strength of personal norms regarding those behaviors (Thøgersen, 2006). Pro-environmental personal norms can influence environmentally friendly actions (Stern et al., 1999). argued that avoiding conflict and maintaining balance are seen as virtues in traditional Asian and collectivist cultures. The higher the focus on interpersonal harmony, the more individuals are calm and content, paying attention to avoiding conflicts and maintaining balance in their interactions. They tend to adjust their personal norms to influence their final behavior (Kahlor, 2007). Those who value interpersonal harmony are more likely to strengthen their environmental norms, thereby promoting environmental actions (Wang et al., 2023).
(4) Ethical evaluation and personal norms have a chained mediating effect between interpersonal harmony and sustainable consumption behavior. Based on VBN theory, this study treats ethical evaluation and personal norms as a chain in the mediation process. This theory is widely used in research on pro-environmental behavior (Zeiske et al., 2021). This study reveals for the first time that interpersonal harmony more easily activates this theoretical path, thereby triggering sustainable consumption behaviors.
(5) Environmental cognition positively moderates the effect of ethical evaluation on personal norms. When consumers perceive themselves as having higher environmental cognition, their ethical evaluations are more likely to be converted into personal norms. This finding aligns with our expectations, as environmental cognition accumulated by consumers increases their perception of environmental responsibility. Consumers with more green knowledge perceive greater value in green consumption and are more capable of social observation (Buenstorf and Cordes, 2008), leading them to prefer environmentally friendly actions (Wang et al., 2021). Individuals with higher levels of environmental cognition generally have a stronger understanding of the severity of environmental issues (Feng and Reisner, 2011), meaning that when their ethical evaluation of sustainable consumption is higher, individuals with greater environmental cognition are more likely to translate this evaluation into personal norms.

### 5.1 Theoretical contributions and implications

Although this study is based on samples from the Chinese market within a Confucian cultural context, its theoretical framework and core mechanisms possess cross-cultural explanatory potential. Chinese consumers, along with global consumers, confront global environmental issues such as climate change and plastic pollution (Lu and Park, 2025). The core theoretical foundations of this study (value-belief-norm theory, moral evaluation, and personal norm mediating mechanisms) have also been validated across diverse cultural contexts.

#### 5.1.1 Theoretical contributions

(1) This study enriches research in sustainable consumption. This study establishes a connection between interpersonal harmony values from a Confucian cultural perspective and sustainable consumption behavior for the first time. It addresses the current reliance on Western cultural value measurement tools, filling a gap in the existing research. It also contributes to the improvement of quantitative research on Chinese local values, particularly Confucian culture. Furthermore, it fills the gap in current research that mainly explains how cultural values influence individual sustainable consumption choices from the perspectives of ecological values and human-nature relationships.
(2) Firstly, it effectively expands the antecedent value variables of the VBN theory, addressing the potential limitation of insufficient predictive power in biosphere values. Secondly, it confirms the VBN theory can also be applied to cross-cultural research. Finally, it further clarifies the chain mediating pathway relationships between upper-level and lower-level variables within the VBN theory, effectively revealing the multiple chain mediating effects of ethical evaluation and personal norms in sustainable consumption behavior.
(3) This study introduces and tests the moderating effect of environmental cognition. This research further improves the theoretical framework for understanding how cultural values and moral ethics influence individual sustainable consumption behavior.

#### 5.1.2 Managerial implications

Sustainable development can be achieved through extensive cooperation among businesses, governments, and consumers (Fan et al., 2015).

(1) China is leading global climate governance with its “dual-carbon” goals (carbon peaking by 2030 and carbon neutrality by 2060), and the findings of this study can provide support for policy tool innovation. Recommendations include integrating interpersonal harmony values into green financial policies and enhancing consumers’ sense of participation through “collective honor incentives” (Ahn et al., 2014). Promoting the advantages of interpersonal harmony and inheriting the excellent cultural traditions of the Chinese people, which include harmonious values, can help maintain stability in organizations, societies, and even countries (Xu et al., 2022).
(2) For manufacturers, fostering the concept of sustainable consumption is essential. Introducing environmental sustainability into business practices is crucial for protecting natural resources and the global ecosystem (Chwialkowska et al., 2024). Leveraging China’s leading platform economy ecosystem (e.g., WeChat, Douyin), develop “low-carbon social” features (such as friend carbon footprint PK) to transform the “social interaction-norm activation” pathway revealed by the research into a traffic economy.
(3) For consumers, the concept of sustainable development should activate their “intergenerational responsibility” through the theme of “family environmental heritage.” Specifically, natural experiences, such as parent–child outdoor early education courses, can raise awareness of sustainable consumption, as outdoor activities that connect with nature enhance both sustainable consumption and overall well-being (Ahn et al., 2014).

### 5.2 Limitations

(1) The study sample was drawn from urban residents in China, specifically from members of large chain supermarkets in major streets in coastal cities. Therefore, the results may not directly apply to consumers in other countries or other types of retail stores.
(2) The study discusses sustainable consumption behavior as a whole. Sustainable consumption includes three dimensions: wisely meeting basic needs, caring for environmental welfare, and considering the needs of future generations. This research does not separately analyse these three dimensions in an empirical study.
(3) In this study, a cross-sectional method is employed for questionnaire processing and analysis, without conducting longitudinal analysis at a specific time period. The questionnaires are self-evaluated by consumers within the same time frame, without separating the measurement sources, time, space, and methods, which may lead to social desirability bias.

### 5.3 Future research directions

(1) Future researchers may consider conducting investigations in other regions, such as the East Asian Confucian cultural sphere (e.g., Japan, South Korea, Vietnam), and may even further undertake cross-national comparative studies. These findings will provide important references for managers to develop marketing strategies targeting cross-cultural consumers with different consumption habits.
(2) Future researchers may consider critically examine the differences between the concept of “interpersonal harmony” and Western constructs within the VBN theory (e.g., altruism). Meanwhile, the field of consumer ethics research is also an important topic in the context of sustainable consumption (Nangia et al., 2024).
(3) Future researchers may employ time series analysis to explore whether research results remain consistent or vary over time (e.g., over six months, one year, etc.), thereby better enhancing the validity of the findings.

## Data Availability

The original contributions presented in the study are included in the article/Supplementary material, further inquiries can be directed to the corresponding author/s.
