# Supplementary material for: The influence of interpersonal harmony on sustainable consumption behavior in China from a Confucian perspective: exploring the dual-path mediating role of ethics and norms
Source: Front Psychol. 2025 Sep 26;16:1664625. doi: 10.3389/fpsyg.2025.1664625 (PMC12511024; doi:10.3389/fpsyg.2025.1664625)
Supplement: Supplementary file 1 [file Supplementary_file_1.pdf]

frontiers

Research on Sustainable Consumption Behavior of Urban Residents

Dear Sir/Madam: Hello!

We are conducting a survey on sustainable consumption behavior of urban residents. Please provide a truthful assessment based on your daily experiences and the items listed in this questionnaire.

Collection Instructions: This survey is for academic research purposes only. All collected data and results will be presented in an anonymous form. We solemnly promise that all your responses will be strictly confidential. Thank you very much for your cooperation!

Scoring Instructions: 1 - Strongly Disagree; 2 - Disagree; 3 - Neutral; 4 - Agree; 5 - Strongly Agree

Appendix I

Survey Scale

Prompt: Based on your feelings, to what extent do you agree with the following descriptions? The larger the number, the higher the degree of agreement. Please mark "○" on the corresponding number.	Degree of Agreement				
	Strongly Disagree	Disagree	Neutral	Agree	Strongly Agree
1. I always try hard to reduce miss-use of goods and services (eg::I switch off light and fan when I am not in the room)	1	2	3	4	5
2. I recycle daily newspaper (eg: use as pet's litter box, etc)	1	2	3	4	5
3. I avoid being extravagant in my purchase	1	2	3	4	5
4. I avoid over use/consumption of goods and services (eg: take print only when needed)	1	2	3	4	5

5. I reuse paper to write on the other side	1	2	3	4	5
6. While dining in restaurant, I order food(s) only the amount that I can eat in order to avoid wasting food	1	2	3	4	5
7. I choose to buy product(s) with biodeg:radable container or packaging	1	2	3	4	5
8. I don't like to waste food or beverage	1	2	3	4	5
9. I recycle my old stuffs in every possible ways (eg: distribute old clothes among needy people)	1	2	3	4	5
10. I reuse shopping bag(s) every time go for shopping	1	2	3	4	5
11. I plan carefully before I purchase product of service	1	2	3	4	5
12. I do care for the natural environment	1	2	3	4	5
13. I use eco-friendly products and services	1	2	3	4	5
14. I purchase and use products which are environmental friendly	1	2	3	4	5
15. I often pay extra money to purchase environmentally friendly product (eg: organic food)	1	2	3	4	5
16. I am concerned about the shortage of the natural resources	1	2	3	4	5
17. I prefer to use paper bag since it is biodeg: radable	1	2	3	4	5
18. I love our planet	1	2	3	4	5
19. I always remember that my excess consumption can create hindrance for the	1	2	3	4	5

future generation to meet up their basic needs					
20. I care for the need fulfilment of the next generation	1	2	3	4	5
21. I often think about future generation's quality of life	1	2	3	4	5
22. I try to control my desire of excessive purchase for the sake of future generation	1	2	3	4	5
23. I am concerned about the future generation	1	2	3	4	5
24. I try to minimise the excess consumption for the sake of preserving environmental resources for the future generation	1	2	3	4	5
25. As a consequence of maintaining harmony and integ:rating differences between people, you are able to broaden your view of the world	1	2	3	4	5
26. Having an ability to interact with others harmoniously is vital for achieving major successes	1	2	3	4	5
27. The idea that interpersonal harmony promotes wealth is a wise one	1	2	3	4	5
28. Maintaining interpersonal harmony is an important goal in life	1	2	3	4	5
29. Making concessions demonstrates your maturity and capacity for forgiveness	1	2	3	4	5
30. Everything prospers when there is harmony in the family; maintaining harmony among family members is very important	1	2	3	4	5

31. If there is no need for forced consensus and everyone has different perspectives, then everyone should be willing to compromise	1	2	3	4	5
32. Being patient and willing to compromise is a show of respect to the other person	1	2	3	4	5
33. It is a virtue to tolerate everything	1	2	3	4	5
34. In interpersonal interactions, you should be considerate of others' difficulties and forgive them whenever possible	1	2	3	4	5
35. Being patient and willing to compromise indicates that a person is gracious and forgiving	1	2	3	4	5
36. Being patient and willing to compromise demonstrates that you have a higher sense of self-discipline than ordinary people	1	2	3	4	5
37. I won't buy environmentally friendly products	1	2	3	4	5
38. Because environmental values are important to me, I feel obligated to take environmental actions	1	2	3	4	5
39. I will take environmental actions based on personal values or principles	1	2	3	4	5
40. When traveling, I will firmly grasp the principles of environmental protection	1	2	3	4	5
41. It is my obligation to conduct pro-environmental behaviors while consuming	1	2	3	4	5

42. It is ethically right to conduct pro-environmental behaviors while consuming	1	2	3	4	5
43. Conducting pro-environmental behaviors while consuming is consistent with my moral principles	1	2	3	4	5
44. Conducting pro-environmental behaviors while consuming is valuable to the sustainable development of tourism sites	1	2	3	4	5
45. Conducting pro-environmental behaviors while consuming can bring positive environmental, ecological and economic benefits	1	2	3	4	5
46. Conducting pro-environmental behaviors while consuming can offer a beautiful natural scenery for future generations	1	2	3	4	5
47. Conducting pro-environmental behaviors while consuming can offer a better living environment for our children and grandchildren	1	2	3	4	5
The following are 5 key environmental issues. You may have a clear understanding of some basic concepts, causes, and related knowledge, while others may be unclear. Please select a number to indicate your level of understanding of each term based on your knowledge of environmental protection: 1 = Very Unfamiliar to 5 = Very Familiar (using a Likert 5-point scale)	Very Unfamiliar	Unfamiliar	Neutral	Familiar	Very Familiar
48. I know that the main cause of global warming is increased carbon dioxide emissions and understand its consequences.	1	2	3	4	5

49. I know that the main cause of sea/river pollution is the direct discharge of industrial wastewater and understand its consequences.	1	2	3	4	5
50. I know that the main cause of pollution of drinking water is the direct discharge of industrial wastewater and understand its consequences.	1	2	3	4	5
51. I know that the main cause of the world population explosion is the rapid growth of population and understand its consequences.	1	2	3	4	5
52. I know that the main cause of acid rain formation is the increased concentration of substances such as sulfur dioxide in the atmosphere and understand its consequences.	1	2	3	4	5

To facilitate our statistical analysis, please provide your basic information (mark "○" on the option that applies or fill in the correct number, etc.).

Your gender: ① Male ② Female

Your age: ① Under 18 ② 18-29 years old ③ 30-39 years old ④ 40-49 years old ⑤ 50-59 years old ⑥ Over 60 years old

Your education level: ① High school/vocational secondary school or below ② Junior college ③ Bachelor's degree ④ Master's degree ⑤ Doctoral degree

Your marital status: ① Unmarried ② Married

The area where you have lived for more than 6 months in the past year: ① Urban area ② Township ③ Rural area

Your current occupation type: ① Government department staff ② Enterprise management personnel ③ General worker or service staff ④ Engineering and technical personnel ⑤ Personnel in the fields of science, education, culture, and health ⑥ Others

Your monthly income falls into which of the following ranges: ① 2,500 yuan and below ② 2,501-5,000 yuan ③ 5,001-7,500 yuan ④ 7,501-10,000 yuan ⑤ 10,001-12,500 yuan ⑥ 12,501 yuan and above
